# Surface Characteristics and Biofilm Development on Selected Dental Ceramic Materials

**DOI:** 10.1155/2017/7627945

**Published:** 2017-05-08

**Authors:** Kyoung H. Kim, Carolina Loch, J. Neil Waddell, Geoffrey Tompkins, Donald Schwass

**Affiliations:** Sir John Walsh Research Institute, Faculty of Dentistry, University of Otago, Dunedin, New Zealand

## Abstract

**Background:**

Intraoral adjustment and polishing of dental ceramics often affect their surface characteristics, promoting increased roughness and consequent biofilm growth. This study correlated surface roughness to biofilm development with four commercially available ceramic materials.

**Methods:**

Four ceramic materials (Vita Enamic®, Lava™ Ultimate, Vitablocs Mark II, and Wieland Reflex®) were prepared as per manufacturer instructions. Seventeen specimens of each material were adjusted and polished to simulate clinical intraoral procedures and another seventeen remained unaltered. Specimens were analysed by SEM imaging, confocal microscopy, and crystal violet assay.

**Results:**

SEM images showed more irregular surface topography in adjusted specimens than their respective controls. Surface roughness (*R*_*a*_) values were greater in all materials following adjustments. All adjusted materials with the exception of Vitablocs Mark II promoted significantly greater biofilm growth relative to controls.

**Conclusion:**

Simulated intraoral polishing methods resulted in greater surface roughness and increased biofilm accumulation.

## 1. Introduction

Dental ceramics are the restorative material of choice for indirect restorations, mainly due to their biocompatibility, low thermal conductivity, color stability, and aesthetics [1]. Dental ceramics are used in restorative dentistry because of their success rate as well as diverse range of chemical and structural compositions, resulting from recent improvements in biomaterial technology [2, 3]. These materials commonly consist of both glassy and crystalline phases, which are usually heat-treated to provide desirable properties [4]. While the glassy phase contributes to the aesthetics of the ceramics [5], the crystalline phase is responsible for the mechanical properties of the material [4].

Structurally, dental bioceramics cover a wide spectrum of glass–ceramics, reinforced porcelains, oxide ceramics (zirconia, alumina, and spinel), fiber-reinforced ceramic composites, and multilayered ceramic structures. In the last two decades, computer-assisted design and manufacturing (CAD/CAM) technologies have replaced the laborious and time-consuming conventional restoration fabrication methods [6]. Two of the new dual network hybrid ceramics or polymer-infiltrated-ceramic-networks are 3M ESPE Lava Ultimate and Vita Enamic. 3M ESPE Lava Ultimate (3M ESPE, USA) is a resin nanoceramic material containing a combination of aggregated 20 nm silica and 4 to 11 nm zirconia clusters in a resin matrix. Vita Enamic (Vita, Germany) is a hybrid ceramic containing 86% feldspathic porcelain and an interpenetrating polymer network 14% by weight. Among glass-based systems or feldspathic porcelains are Vitablocs Mark II and Wieland Reflex. Vitablocs Mark II (Vita, Germany) is a fine-grained, homogeneous feldspathic porcelain with an average particle size of 4 *μ*m. Wieland Reflex is a feldspathic porcelain which contains homogenous nanoleucite crystals [7–9].

Fixed prostheses such as ceramic crowns often require adjustments before and after cementation, which generally removes the smooth external glazed surface layer [10]. The exposed porcelain layer is commonly roughened in comparison to the normal smooth glazed surface. In addition, abrasive machining processes in CAD/CAM systems often induce damage, generating the need for final finishing in oral conditions using a dental handpiece and diamond burs [6]. Surface roughness, a component of surface texture, influences how a material interacts with the environment [11]. A higher degree of surface roughness of ceramic restorations often generates greater wear on the opposing dentition, compromises aesthetics of the restoration, and increases biofilm adhesion and growth [12].

Microscopic irregularities caused by cracks, grooves, and abrasion defects often lead to greater surface roughness, becoming common sites for bacterial adhesion [13]. These surface irregularities provide shelter for bacteria from shear forces generated in the oral cavity, allowing them to form stronger bonds to the substratum [14]. Greater biofilm formation on dental surfaces has been implicated in the development of both gingivitis and dental caries [15]. For these reasons, material predilection to biofilm formation is an important consideration in the selection of restorative materials.

Most evidence supporting clinical use of newer ceramic materials is from information provided by manufacturers regarding the chemical composition and physical properties (e.g., [16–18]). Information on surface characteristics and biofilm formation on new materials is sparse, particularly with respect to the impact of intraoral polishing.

The aims of this study were to characterize the surface ultrastructure and roughness of four ceramic materials and to assess their promotion of biofilm development following adjustments simulating clinical intraoral polishing.

## 2. Material and Methods

### 2.1. Specimen Preparation

Four ceramic materials (Table 1) were studied. Each of the CAD materials was sectioned into rectangular blocks with a diamond disc (Komet 918B, Komet Dental, Germany) attached to a straight handpiece. The veneering porcelain material (Wieland Reflex veneering porcelain) was formed into disks using a stainless steel mould and fired (Dekema Austromat M, Dekema, Germany), followed by further glaze firing according to manufacturer guidelines. Thirty-four specimens of variable dimensions were prepared for each material, being ultrasonically cleaned for 10 min and air dried, and the surfaces were polished following manufacturer guidelines. The number of specimens analysed was determined taking into account material availability and preparation constraints.

Seventeen specimens of each ceramic material were adjusted and polished to simulate clinical intraoral procedures. The polishing sequences were carried out using a Powertorque Lux 646B high speed handpiece (KaVo, Biberach, Germany) under water irrigation. Adjustments used a “rugby ball” diamond bur, followed by polishing with ISO standard grit size red (30 *μ*m), yellow (15 *μ*m), and white (8 *μ*m) fine finishing burs (B260, B261, and B262). Final polishing used an extrafine porcelain bur (Cerapol Plus, Edenta, Hauptstrasse, Switzerland). The other 17 specimens of each material were unaltered to use as controls. Physical dimensions of each specimen were measured in order to calculate the surface areas.

### 2.2. Surface Characterization

One control and one adjusted specimen of each ceramic material were coated with gold palladium for scanning electron microscopy (SEM) observation. SEM images were obtained in a Field Emission SEM (JSM-6700F, JEOL Ltd., Tokyo, Japan) operating at 10 kV and 10 *μ*A. Magnifications ranged from 25x to 2000x. Material topography was evaluated through qualitative assessment of the surface characteristics of different materials observed via SEM imaging.

### 2.3. Surface Roughness

One further control and one adjusted specimen of each material were examined under a confocal laser-scanning microscope (LSM 510 Upright Axioplan, Carl Zeiss Inc., Oberkochen, Germany). Stacks of confocal microscope images were edited using 3D Slicer [19] to level the images which were analysed using Fiji and additional plugins including “extended depth of field” and “surfaceChJ 1q” (ImageJ, NIH, Bethesda, Maryland, USA). Height maps were generated, and peak-to-valley surface roughness (*R*_*a*_) values were measured at three spots for each specimen at randomly selected locations and averaged.

### 2.4. Biofilm Growth

The remaining control and adjusted specimens of each material were placed in the wells of a tissue-culture tray (CELLSTAR® 6-well, Greiner Bio-one, Kremsmünster, Austria). Specimens were embedded in polyvinyl siloxane (PVS) impression material, leaving only the prepared surfaces exposed to biofilm growth. The trays were then irradiated under UV light in a laminar flow cabinet for 15 min.

The bacterial strain* Streptococcus gordonii* C219 (from the University of Otago culture collection) was grown for 24 h in 10 ml of brain heart infusion (BHI; BD Biosciences, Franklin Lakes, United States) and 30 *μ*L aliquots used to inoculate each well of the specimen-containing wells, which each contained BHI (7.5 mL). Culture trays were incubated for 72 h at 37°C and the medium was replaced every 24 h. Specimens were removed from the impression material, rinsed thoroughly with phosphate buffered saline (PBS) on an orbital shaker (42 rpm for 15 minutes), and placed into new culture trays. Crystal violet (0.1% in 25% methanol) was added (5 mL) to each well and after 15 minutes, the trays with embedded specimens were washed (8 L of water per tray), avoiding direct application onto the specimen. The trays with specimens were dried overnight.

The unprepared surfaces of each specimen were coated with resin and light-cured to prevent crystal violet solubilization because biofilm growth also occurred on the unexposed surfaces despite covering with PVS. Sealing the unexposed surfaces eliminated possible effects of this growth on the assessment of the test surface biofilm. Acetic acid (3 mL at 30%) was added to each well to elute the crystal violet from the biofilm on the exposed surfaces. The adsorption (*A*_570_) of the eluted stain was determined using an Ultrospec 6300 Pro (Amersham Biosciences, Buckinghamshire, UK), as a measure of relative biomass. Due to the different sizes of the specimens analysed, absorbance values (*A*_570_) obtained were then standardised by dividing *A*_570_ values by the surface area of each specimen.

### 2.5. Statistical Analyses

The nonparametric Mann–Whitney* U* Test for two independent samples was employed to test differences in the biofilm biomass between pairs of adjusted versus control surfaces for each material. Statistical significance was set at the 5% probability level. Tests were performed with BioStat 2009 (AnalystSoft, Alexandria, VA, USA).

## 3. Results

### 3.1. Surface Characterization by SEM

**Figure 1:**
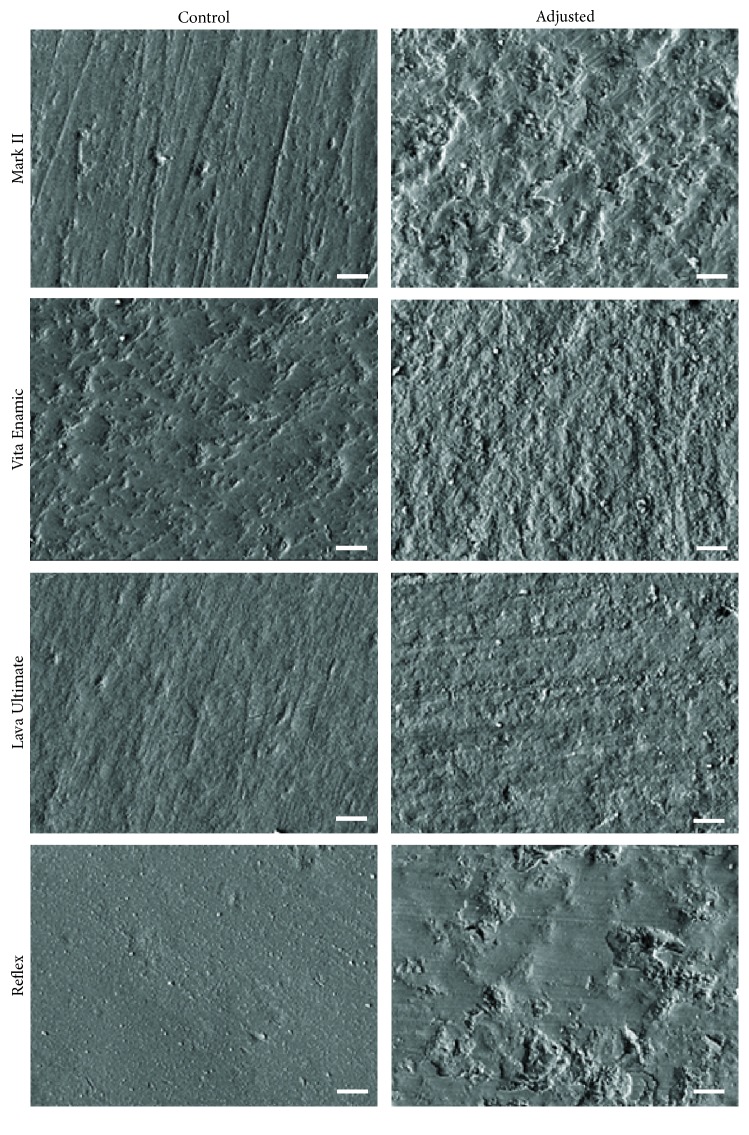
SEM images (magnification 1000x) of control and adjusted surfaces of ceramic materials (scale bar = 10 *μ*m).

For all materials analysed, SEM images revealed that the surfaces which were adjusted and polished to simulate clinical intraoral procedures had more irregular surface topography than the respective controls (Figure 1). Parallel scratch marks and small pits were more often seen on control specimens, which was consistent with normal preparation and polishing artefacts. Specimens which were adjusted and polished displayed coarse pits and irregularities, as evidenced by a rougher surface. The control and adjusted Vitablocs Mark II specimens exhibited the roughest surfaces, whereas Wieland Reflex glazed porcelain recorded the smoothest control surfaces, and 3M ESPE Lava Ultimate had the smoothest adjusted surfaces, with relatively small pits and scratches.

### 3.2. Surface Roughness

**Figure 2:**
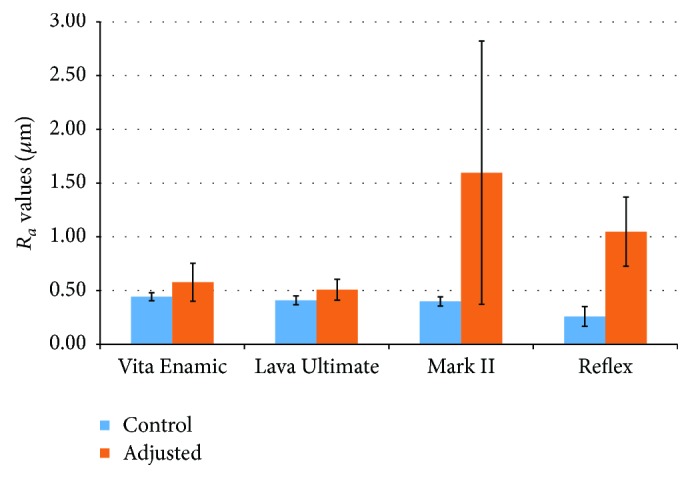
Surface roughness (*R*_*a*_) of control and adjusted surfaces of ceramic materials (average ± SD).

*R*_*a*_ values were higher for all adjusted surfaces in comparison to controls (Figure 2). For both hybrid ceramics (Vita Enamic and 3M ESPE Lava Ultimate) adjustment resulted in minor increases in *R*_*a*_ values in comparison to controls. Conversely, adjusted surfaces of Vitablocs Mark II and Wieland Reflex porcelain showed much higher *R*_*a*_ values than controls. The highest *R*_*a*_ values were recorded for the adjusted Vitablocs Mark II specimen (1.597 *μ*m ± 1.22), while the lowest was the control Wieland Reflex porcelain (0.260 *μ*m ± 0.09). Measures of surface roughness (*R*_*a*_ values) were not tested statistically due to the small sample sizes available for this analysis.

### 3.3. Biofilm Development

**Figure 3:**
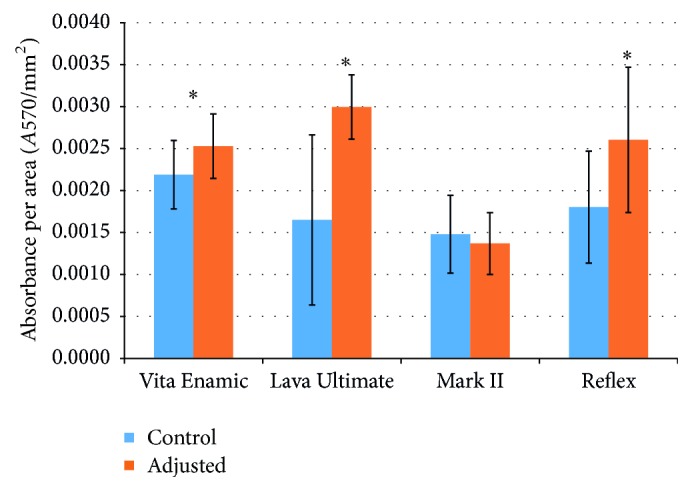
Comparison of biofilm development on control and adjusted surfaces of ceramic materials (average ± SD). ^*∗*^Statistically significant (*p* < 0.05, 95% confidence interval).

With the exception of Vitablocs Mark II, adjustment of the surface promoted significantly greater biofilm growth: Lava* U* = 216.5, *p* < 0.001; Vita Enamic* U* = 168, *p* = 0.021; Wieland Reflex* U* = 183.5, *p* = 0.003 (Mann–Whitney* U* test, 95% confidence interval). Surface adjustment of Vitablocs Mark II did not influence biofilm accumulation (*U* = 92.5, *p* = 0.407). Adjusted 3M ESPE Lava Ultimate promoted the greatest biofilm accumulation (0.003 ± 0.0004 *A*570/mm^2^), while adjusted Vitablocs Mark II was least supportive of biofilm development (0.0014 ± 0.0004 *A*570/mm^2^) (Figure 3).

## 4. Discussion

This study explored the impact of simulated intraoral adjustment and polishing procedures on surface ultrastructural characteristics, surface roughness, and biofilm growth for four commercially available ceramic materials, including two new hybrid materials. For most ceramic materials, finishing or contouring involves adjustments required to achieve the final shape of the restoration, generally using ultrafine diamonds and/or coarse rubber abrasives. Polishing is required to achieve the final surface smoothness of the restoration with minimal changes to surface shape [9].

SEM indicated that adjusted surfaces had more surface irregularities compared to control surfaces for all materials analysed and that these features were dependent on the specific material, reflecting the diverse chemical compositions and varied fabrication methods. 3M ESPE Lava Ultimate, Vita Enamic, and Vitablocs Mark II were prefabricated CAD/CAM blocks, presenting more regular surfaces than Wieland Reflex. These machinable ceramics may involve less volume defects than the laboratory-fabricated Wieland Reflex porcelain [20]. It seems that if intensive intraoral polishing procedures are utilized, then restorations prepared from CAD/CAM blocks would be expected to have a smoother finish than those of adjusted surfaces of other ceramics, such as the Wieland Reflex porcelain. Surface porosities and irregularities cannot be easily eliminated by conventional intraoral polishing processes [12, 21].

In accordance with our findings, studies of intraoral polishing methods generally report increases in the roughness of ceramic surfaces [22–24], but there is not yet a consensus. Reports are inconsistent because of different measuring parameters, combinations of polishing methods, and the variety of tested ceramic materials [25]. Glazed microfilled feldspar ceramics present less surface roughness than do materials submitted to glaze followed by diamond bur polishing. However, when ceramic surfaces are treated with a bur and polished with rubber tips, roughness values decrease [23]. Our study revealed that all materials tested had greater *R*_*a*_ values following polishing adjustments. In particular, adjustment of Vitablocs Mark II and Wieland Reflex porcelain increased *R*_*a*_ values by 1.197 *μ*m and 0.789 *μ*m, respectively. For the hybrid ceramics Vita Enamic, and 3M ESPE Lava Ultimate, the increases in *R*_*a*_ values were not as pronounced.

Fasbinder and Neiva [9] report that a resin nanoceramic material (3M ESPE Lava Ultimate) is smoother than hybrid ceramic (Vita Enamic), and both are smoother than glazed-fired leucite-reinforced ceramics. Manually polished CAD/CAM materials are often smoother than conventional ceramics after either polishing or glazing [9]. Flury et al. [26] also evaluated 3M ESPE Lava Ultimate and Vita Enamic by roughening specimens in a standardised manner followed by polishing with three different systems. Surface roughness and microhardness were measured immediately after polishing and after six-month storage with monthly artificial tooth brushing. The surface of Vita Enamic was less affected by storage and tooth brushing despite lower overall *R*_*a*_ values for 3M ESPE Lava Ultimate [26].

Studies aiming at developing optimal finishing techniques report surface roughness values as low as 0.171 *μ*m [27]. However, this involves intensive polishing procedures incorporating diamond pastes, whereas our study constrained polishing to simulate clinical conditions, resulting in greater roughness.

The effect of surface roughness on biofilm growth has been assessed by both in vitro and in vivo assays [14, 24, 28, 29]. In the current study, with the exception of Vitablocs Mark II, adjustment resulted in increased biofilm development on all surfaces, which accords with a previous report [22]. Though it is difficult to determine the relative contributions, surface roughness, surface free energy, and chemical composition all influence biofilm development [14, 22, 23, 28, 30].

Decreasing abrasive machining-induced surface and subsurface damage in CAD/CAM and intraoral finishing processes is a major challenge to bioceramics engineering. Surface roughness can negatively influence ceramic strength as brittle cracks can lead to either earlier or catastrophic failure of enamel and prosthetic components [31]. In addition, roughened surfaces increase abrasion to antagonistic tooth structures [6, 25]. On the other hand, polishing could improve not only aesthetic surface texture but also the strength of ceramics, due to the elimination of surface flaws and development of residual compressive stresses in the porcelain surfaces [6].

Rough surfaces facilitate the adhesion of bacteria by providing niches where bacteria can adhere and grow protected from brushing, muscular action, and salivary flow [23]. Commonly employed finishing/polishing procedures have clinical relevance, particularly when procedures are conducted close to the cervical area of indirect restorations, where biofilm formation can result in either dental caries or periodontal disease [23].

For both surface characterization via SEM and surface roughness analyses, only one specimen per group was used. Although it is expected that the features described here were typical of each material, the results were indicative and the conclusions were made with caution. In addition, this in vitro study used a monoculture biofilm, whereas oral biofilms are complex communities of microorganisms [32]. This investigation also did not account for the influences of saliva, temperature, and pH changes on biofilm growth. Despite these limitations, this study demonstrated that simulated intraoral polishing methods resulted in greater surface roughness and increased biofilm growth for the four materials investigated. Future studies in a clinical setting will further elucidate the significance of these findings.

## Figures and Tables

**Table 1:** Ceramic materials tested in this study.

Product	Type	Manufacturer
Vita Enamic	Hybrid ceramic	Vita Zahnfabrik, Bad Säckingen, Germany
3M ESPE Lava Ultimate	Hybrid ceramic	3M ESPE, Minnesota, USA
Vitablocs Mark II	Leucite-reinforced glass ceramic	Vita Zahnfabrik, Bad Säckingen, Germany
Wieland Reflex veneering porcelain	Nanoleucite-reinforced glass ceramic	Wieland Dental, Pforzheim, Germany

## Additional Points

*Practical Implications*. Intraoral adjustment and polishing of dental ceramics may result in greater surface roughness which can contribute to biofilm adhesion and growth.
